# Research on the Vision-Based Dairy Cow Ear Tag Recognition Method

**DOI:** 10.3390/s24072194

**Published:** 2024-03-29

**Authors:** Tianhong Gao, Daoerji Fan, Huijuan Wu, Xiangzhong Chen, Shihao Song, Yuxin Sun, Jia Tian

**Affiliations:** College of Electronic Information Engineering, Inner Mongolia University, Hohhot 010021, China; 32156037@mail.imu.edu.cn (T.G.); wuhuijuan@imu.edu.cn (H.W.); 32156032@mail.imu.edu.cn (X.C.); 32156060@mail.imu.edu.cn (S.S.); 32156020@mail.imu.edu.cn (Y.S.); 32156068@mail.imu.edu.cn (J.T.)

**Keywords:** dairy cows, ear tag detection, ear tag recognition, Small-YOLOV5s

## Abstract

With the increase in the scale of breeding at modern pastures, the management of dairy cows has become much more challenging, and individual recognition is the key to the implementation of precision farming. Based on the need for low-cost and accurate herd management and for non-stressful and non-invasive individual recognition, we propose a vision-based automatic recognition method for dairy cow ear tags. Firstly, for the detection of cow ear tags, the lightweight Small-YOLOV5s is proposed, and then a differentiable binarization network (DBNet) combined with a convolutional recurrent neural network (CRNN) is used to achieve the recognition of the numbers on ear tags. The experimental results demonstrated notable improvements: Compared to those of YOLOV5s, Small-YOLOV5s enhanced recall by 1.5%, increased the mean average precision by 0.9%, reduced the number of model parameters by 5,447,802, and enhanced the average prediction speed for a single image by 0.5 ms. The final accuracy of the ear tag number recognition was an impressive 92.1%. Moreover, this study introduces two standardized experimental datasets specifically designed for the ear tag detection and recognition of dairy cows. These datasets will be made freely available to researchers in the global dairy cattle community with the intention of fostering intelligent advancements in the breeding industry.

## 1. Introduction

Milk is an essential nutrient in human life, as it improves people’s food structure and enhances people’s physical fitness. The dairy industry is an important measure of the level of development of national animal husbandry. With the return of the economy to a period of growth and with the improvement of people’s quality of life, the demand for products from the dairy industry has grown more and more, thus leading to the rapid development of dairy farming [1]. In recent years, the scale of dairy farming in China has gradually expanded; the growth of medium and large dairy farms with more than 500 heads accounted for a gradual increase. Despite the rapid development of dairy farming in China, there are still many problems, such as the rough refinement of management; the low degree of information and automation is still an important obstacle to the development of dairy farming [2]. Scientific farming, precision farming, and professional farming are the main developmental directions through which to improve the efficiency of modern farming [3]. The individual identification of dairy cows is the basis of digital farming and precision farming, and it is of great significance for individual positioning, loss tracking, health monitoring, behavioral analysis, and the improvement in the level of modern management of dairy farming and its economic efficiency.

Ear tags serve as the exclusive means of identification for distinguishing individual cows, and their usage is widespread. In large-scale dairy farms, cows are equipped with plastic shovel-shaped ear tags, which consist of a main face, neck, head, auxiliary face, and locking buckle. The predominant color of these tags is yellow, with occasional variations in green, red, and blue. Each ear tag is marked with a numerical identifier, which is handwritten, printed, or characterized by a combination of both styles. The identifier includes the cow’s date of birth and a three- to six-digit ID number.

At present, there are two main ways of distinguishing individual cows by recognizing their ear tag numbers. One is manual identification, which requires a large amount of human effort; as the numbers are difficult to identify, this method is not adapted to the development needs of modern dairy farming. The other method is that of automatic ear tag number recognition based on deep learning and traditional image processing technology.

This method currently faces many technical difficulties, such as the following: (1) The recognition target is tiny and moves randomly, including due to various behavioral postures, making it impossible to directly recognize using static scene text recognition methods. (2) The recognition scene is complex, with ear tags often obstructed due to railings, cow hair, and other elements. Moreover, an image may contain a large number of ear tags, each with diverse colors and angles. (3) The fonts on ear tags include both handwritten and printed styles, with some ear tags featuring both on the same tag. The handwriting styles vary widely, and each ear tag can contain one to three lines of numeric text, posing a significant challenge to the model’s recognition accuracy.

To address the aforementioned challenges, and in comparison with various methods proposed in the current relevant literature, the main innovations and contributions of this paper are outlined as follows:

- A lightweight Small-YOLOv5s, specifically designed for small object detection, is proposed for direct application in dairy cow ear tag detection. It improves the detection accuracy and speed while significantly decreasing the number of parameters and computational overhead, and its hardware deployment to meet the real-time requirements of practical application scenarios is easy. The model is robust to light, tilt, occlusion, and other types of interference, thus overcoming the existing limitations concerning the use of a single color in ear tag detection with traditional image processing techniques, and it can simultaneously detect ear tags of various colors with multiple poses of dairy cows, unaffected by background color interference.
- The ear tag recognition method using DBNet [4] combined with CRNN [5] can quickly and accurately locate and recognize a multi-line regions of text on the same ear tag. Preprocessing operations such as character-level segmentation and image orientation correction are avoided, and images of handwritten and printed numbers on ear tags are mixed into the model during the model training stage so that it has a high recognition accuracy and generalization ability for numbering in the two types of fonts, as well as distortion, deformation, and other irregularities.
- Two sets of annotated experimental datasets in standardized format are released for ear tag detection and ear tag recognition; they are not limited to these two uses, and they can also be used for individual cow detection, behavioral recognition, and the training of other text recognition models. Currently, there is no unified and standardized experimental dataset for cow ear tag identification; in order to promote the modernization and development of dairy farming, the datasets in this study are free and open to the world.

This paper is organized as follows: Section 2 presents a literature review related to individual cow identification, Section 3 presents the experimental dataset for ear tag detection and ear tag recognition, Section 4 details the research methodology of this study, Section 5 presents the experimental results and analysis, and, finally, in Section 6, the study is concluded, and outlooks are provided.

## 2. Related Work

At present, according to different farm sizes, management objectives, and needs, the individual identification of dairy cows can be divided into three main methods: artificial mechanical identification, contact electronic identification, and image biometrics [6].

Artificial mechanical identification can be broadly divided into two methods; one involves making permanent specific markings on the body of a cow. According to the different needs for the identification of corresponding individuals, this is done manually, mainly via carving the ears and branding. Ear engraving involves cutting out different numbers of notches on a cow’s ear to identify the corresponding group or individual cows [7]. Branding methods can be divided into electric branding and freeze branding; electric branding involves using a branding iron to print a number on different parts of a cow’s body, and freeze branding involves implanting a number of different pigments into a cow’s body using a freezing method [8]. Another method of manual identification is the manual viewing of ear tag numbers, through which cows are given ear tags with numbers, and then individual identification is performed by manually viewing the ear tag numbers [9]. Although artificial mechanical identification methods are simple and easy to implement, they consume significant labor costs, the rate of effective identification is low, and they cause some damage to the cattle, which is not suitable for large-scale breeding at modern pastures.

Contact electronic identification technology can be divided into radio frequency identification technology and wearable wireless sensor technology. Radio frequency identification technology involves cows wearing RFID tags as a signal source to transmit a certain frequency of signals, which are received by a tag reader; then, they are sent to a management server and to the user. The forms of tagging are mainly ear tags, injections, and capsules [10]. Wearable wireless sensor technology is more commonly used, and it involves cows wearing collars and leg rings fitted with specific sensors [11]. This method can record a large amount of information about individual cows, which improves the identification efficiency and accuracy, but there are certain security risks, such as the ease of tampering with the tags, their susceptibility to network transmission errors, the vulnerability of the server to network security attacks, and, at the same time, the high equipment cost, which requires professionals to wear and maintain them to address their poor compatibility and weak adaptability.

Currently, biological image recognition techniques can mainly be divided into two kinds: One is the identification of cows based on their own characteristics, such as through cow face recognition and individual detection recognition based on body hair color patterns; the second is the identification of cows wearing specific numbered tags and then using computer vision and image processing technology to identify the tag numbers, such as through ear tag identification and neck ring identification [12]. Qiao et al. [13] proposed a cow face recognition method by first extracting the cows’ facial features using a CNN and then combining this with LSTM to recognize cows’ faces; the method’s recognition accuracy reached 88%. Tassinari et al. [14] implemented video-based cow body detection based on cow color pattern features using YOLOv3 with an average accuracy of 66%. Chen et al. [15] proposed the detection of cow bodies using Mask R-CNN and then VGG16 for individual recognition, and the recognition accuracy was 85.4%. Hao et al. [16] proposed YOLOv5-EMA, a model for the detection of the bodies and vital parts of cows in complex contexts. Zhang et al. [17] introduced a method for recognizing cow neck rings. Initially, they employed a cascade detector with a gradient-based histogram of direction features and a multi-angle detection approach for accurate neck ring localization. Subsequently, they applied image binarization for character segmentation. Finally, a CNN was utilized for character recognition, resulting in a final detection accuracy of 96.89% and a character recognition accuracy of 93.35%.

Ilestrand [18] proposed a cow ear tag recognition method. Initially, they applied a color threshold method in combination with flood filling and projection methods for the detection and segmentation of cow ear tags. Subsequently, traditional optical character recognition methods, such as template matching, K-neighborhood, and a support vector machine, were employed to identify the ear tag numbers. Zin et al. [19] first utilized YOLO for cow head detection, followed by employing a color threshold method and flood filling for ear tag detection and segmentation. They then applied image processing techniques for character segmentation and employed a CNN model for character recognition, achieving a recognition accuracy of 92.5%. John et al. [20] used a color threshold method for direct ear tag detection from images. They corrected the segmentation of ear tags using image processing techniques and employed a CNN for character recognition. In these methods, the use of the color threshold method for detection encountered a limitation, as it only detected a specific color target after setting the color threshold, excluding other colors of ear markers. Additionally, all of these methods required character-level segmentation, resulting in slower recognition speeds.

By analyzing the current research on methods related to individual cow recognition, in order to achieve low-cost and non-invasive individual cow recognition and improve the recognition accuracy in large-scale farming against a complex background, we proposed a vision-based automatic recognition method for cow ear tags with a self-constructed dataset. The experimental results show that the recognition accuracy is high and meets the practical application requirements.

## 3. Dataset

The experimental data presented in this study were gathered from a dairy farm located in Ordos, Inner Mongolia, China, focusing on Simmental cows as the experimental subjects. The dataset comprised two distinct sets, namely, “CEID-D” and “CEGD-R”. The CEID-D dataset was specifically employed for ear tag detection experiments, while the CEGD-R dataset was utilized for ear tag recognition experiments. Both experimental datasets underwent meticulous labeling using an annotation tool and were saved in a standardized format. These datasets have been made freely accessible to the global community through the following URLs: https://www.kaggle.com/datasets/fandaoerji/cow-eartag-detection-dataset (accessed on 20 January 2024) and https://www.kaggle.com/datasets/fandaoerji/cow-eartag-recognition-dataset (accessed on 20 January 2024).

### 3.1. Cow Ear Tag Detection Dataset (CEID-D)

**Figure 1:**
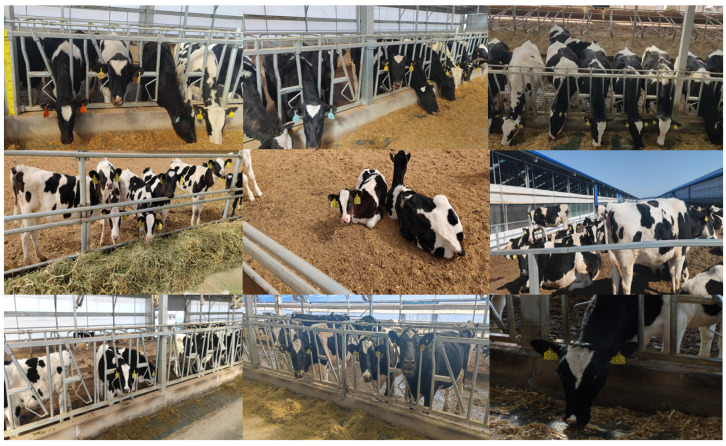
Some samples of data from CEID-D. Capture angles: frontal, lateral, and overhead views of cows. Weather conditions during shooting: overcast and sunny days. Captured cow poses: standing, feeding, and lying down.

The CEID-D dataset was captured using a 2-meter-high tripod or a handheld 2-megapixel camera at a distance of 3–5 meters from the cows; images were captured with a resolution of 4000 pixels (horizontal) × 2200 pixels (vertical), and video data were recorded at a frame rate of 25 frames/s. The dataset encompasses diverse scenarios, including both indoor and outdoor environments, varying lighting conditions, different weather situations, multiple shooting angles, and various behavioral postures of cows. Additionally, it portrays a densely populated environment in the distribution of cows. This is shown in Figure 1.

This dataset consisted of two folders, namely, “Images” and “Labels”. Within the “Images” folder, there were 2675 cow images, which were named from “cow0.jpg” to “cow2674.jpg”. The “Labels” folder contained 2675 TXT-format label files, which were named from “cow0.txt” to “cow2674.txt”. These label files were generated using the Labelimg data annotation tool to annotate the ear tag areas of the cows.

Each label file contained one or more lines of text information, with each line corresponding to the category and location details of each ear tag region. Since this study focused on detecting only the ear tag category, the first digit “0” in each line represented the ear tag category. The location information of the ear tag region was represented with four data points ($\left[x_{\mathrm{center}},y_{\mathrm{center}},h,w\right]$), indicating the normalized center point coordinates of the annotated ear tag rectangle, along with the width and height, respectively.

### 3.2. Cow Ear Tag Recognition Dataset (CEGD-R)

**Figure 2:**
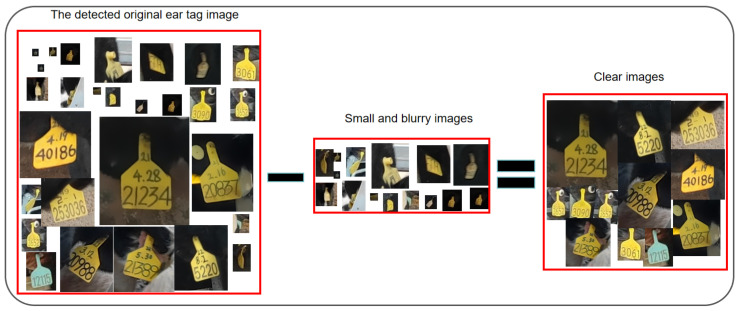
Ear tag image quality assessment.

The CEGD-R is an experimental dataset for ear tag recognition made by cropping the ear tag images detected with Small-YOLOV5s after quality assessment and preprocessing operations. Due to some of the ear tag images obtained through detection being small in size and blurry, making it difficult for the human eye to discern the ear tag numbers, they could not be annotated. Consequently, this study employed a specific image quality assessment mechanism to filter out images that were either unrecognizable or had ear tag numbers partially obscured from the 9705 images detected. As a result, 3238 images that were relatively clear and complete were retained for the creation of the ear tag recognition dataset, as illustrated in Figure 2.

**Figure 3:**
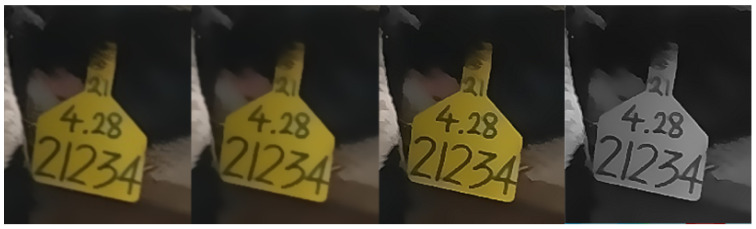
Preprocessing of ear tag images. From left to right, the original ear tag, the ear tag after bilateral filtering, the ear tag after edge sharpening, and the ear tag after grayscaling.

The image quality assessment mechanism initially eliminated ear tag images smaller than 40 × 40 pixels, based on certain empirical values, and then the Laplace transform used to calculate the fuzzy degree; the images with a fuzzy degree of less than 30 were filtered out according to empirical values, and the clearer and more complete ear tag images were retained through manual filtering; finally, the filtered images were used as the experimental data for ear tag recognition after the preprocessing operations of bilateral filtering, edge sharpening, and graying, as shown in Figure 3.

This dataset also comprised two folders, namely, “Images” and “Labels”. The “Images” folder held 3238 images of the processed grayscale ear tags described above with a mix of handwritten or printed numbers, and they were named from “eartags0.jpg” to “eartags3237.jpg”. The “Labels” folder contained 3238 TXT-format label files, which were named from “gt_eartags0.txt” to “gt_eartags3237.txt”. These labels were generated using the Paddlelabel annotation tool to annotate the numbered areas on the ear tags.

**Figure 4:**
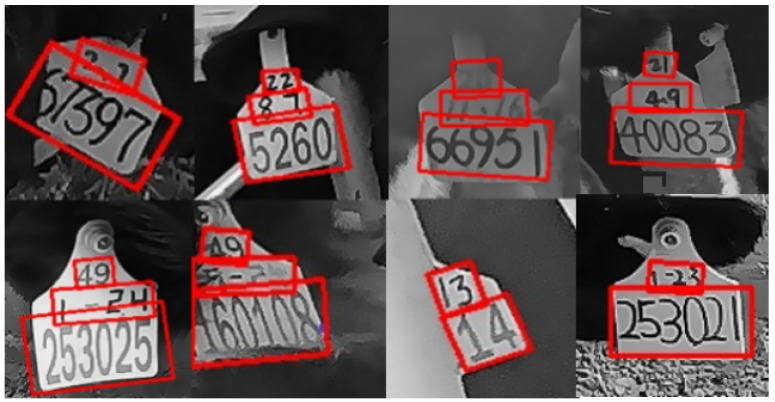
Ear tag images annotated with Paddlelabel.

Each label file consisted of one to three lines of varying text information corresponding to the position details and specific content of the text on the respective ear tag. The position information was provided by the first eight data points, which represented the coordinates of the four endpoints of each numbering area. Each coordinate was expressed as (x, y), and the last data point signified the actual content within the annotated box. Partial data annotated with Paddlelabel are illustrated in Figure 4.

## 4. Method

**Figure 5:**
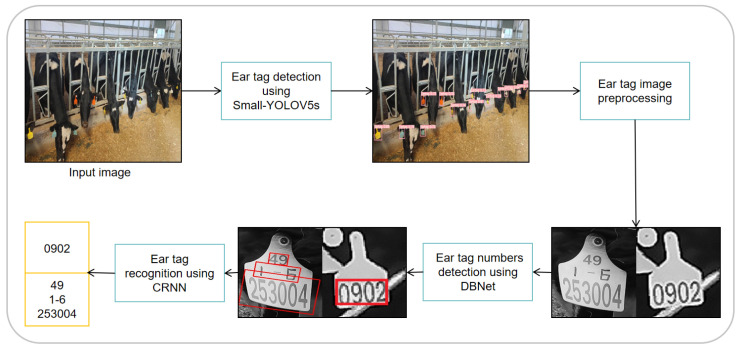
Technology Roadmap.

This study mainly consisted of three parts: cow ear tag detection, ear tag number detection, and ear tag recognition, as illustrated in Figure 5. Firstly, a lightweight Small-YOLOV5s model was employed to directly perform cow ear tag detection on input images. Subsequently, the detected ear tag images underwent preprocessing and were subjected to number region localization using DBNet. Finally, the ear tag numbers were recognized using a CRNN.

### 4.1. Ear Tag Detection

**Figure 6:**
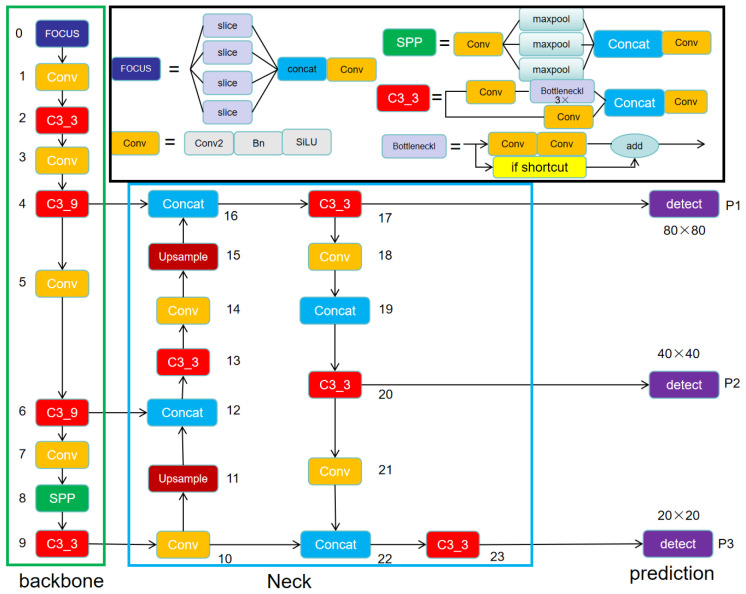
The structure of YOLOV5s.

Due to the excellent detection performance, fast inference speed, and small model size of YOLOv5s [21], meeting the real-time requirements and accuracy of dairy cow ear tag detection, we adopted YOLOv5s as the basic network structure for ear tag detection. The model architecture is illustrated in Figure 6 below.

**Figure 7:**
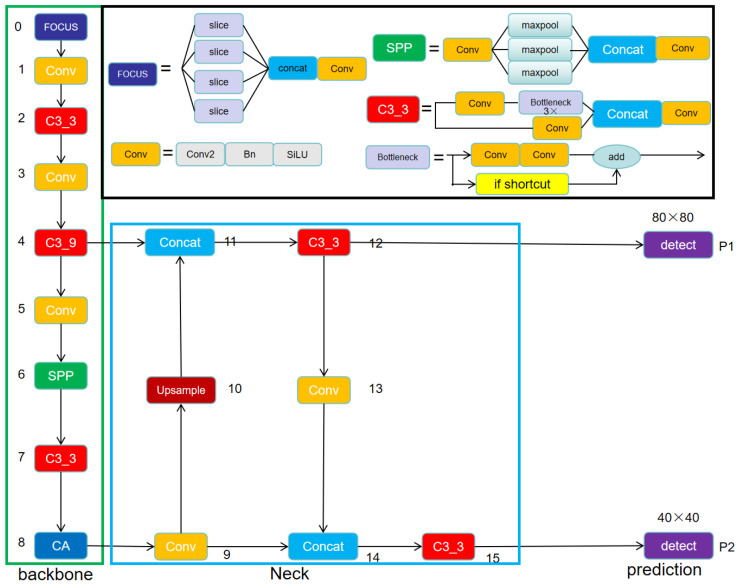
The structure of Small-YOLOV5s.

In the captured raw images, the dimensions of each dairy cow’s ear tag region are relatively small, with an area less than 0.2 of the entire image. Consequently, the detection of dairy cow ear tags is classified as small object detection [22]. In order to enhance the speed and accuracy with which YOLOV5s detects small objects, we proposed an improvement to the YOLOV5s model named Small-YOLOV5s. It consisted of three parts: Backbone, Neck, and Prediction. The model’s structure is illustrated in Figure 7.

The Small-YOLOv5s model employs the Focus slice operation, C3 module, and standard convolution (Conv) modules in its Backbone to extract features from the input image. The Neck uses upsampling and Conv to form a PANet (Path Aggregation Network) [23], enabling bidirectional feature fusion from the top down and bottom up across the Backbone’s feature maps, outputting two feature maps with resolutions of 80 × 80 and 40 × 40 to the Prediction part. The Prediction part, through bounding box regression and parameter iteration during network training, detects objects on these feature maps.

Compared to YOLOV5s, Small-YOLOV5s significantly reduced the model parameters, accelerated the detection speed, and reduced the computational overhead while improving the detection accuracy. The improvements included the following two points.

- **Reduction in the number of convolutional layers**

The Backbone of YOLOV5s primarily employed convolution operations to extract features from the input image, transforming it into differently sized feature maps with strong positional and semantic information. As the convolution layers deepened, the resolution of the feature maps decreased, with stronger semantic information and weaker positional information. Considering the relatively simple structure of the cow ear tags, less semantic information was required, but there was more of a focus on the positional information of the ear tag region, and higher-resolution feature maps are more effective for small object detection. Therefore, we removed the C3_9 module on the sixth layer and the Conv module on the seventh layer from the original YOLOV5s model’s Backbone. Due to the removal of these two modules, the output channel of the Backbone changed from 1024 to 512, and there was a reduction in the number of layers in both the Neck and Prediction parts. The Neck part directly performed upsampling and feature fusion with the fourth layer after the ninth layer, reducing the number of layers from 14 to 7.

The feature map resolution of the last detection layer in Prediction was increased from 20 × 20 to 40 × 40, and the feature maps were reduced from three types (80 × 80, 40 × 40, and 20 × 20) to two types (80 × 80 and 40 × 40), correspondingly eliminating one detection layer (P3). A smaller feature map resolution resulted in a larger receptive field, mapping a larger area of the original-sized image and making it more suitable for predicting larger targets. Conversely, a larger feature map resolution was more suitable for small object detection. For cow ear tag detection, this study proposes an improvement by reducing the depth of the convolution layers, removing unnecessary detection layers for large targets, and retaining layers suitable for medium and small object detection. This not only significantly reduced the model parameters and improved the inference speed but also enhanced the detection performance for small objects.

- **Addition of coordinate attention (CA)** [24]

**Figure 8:**
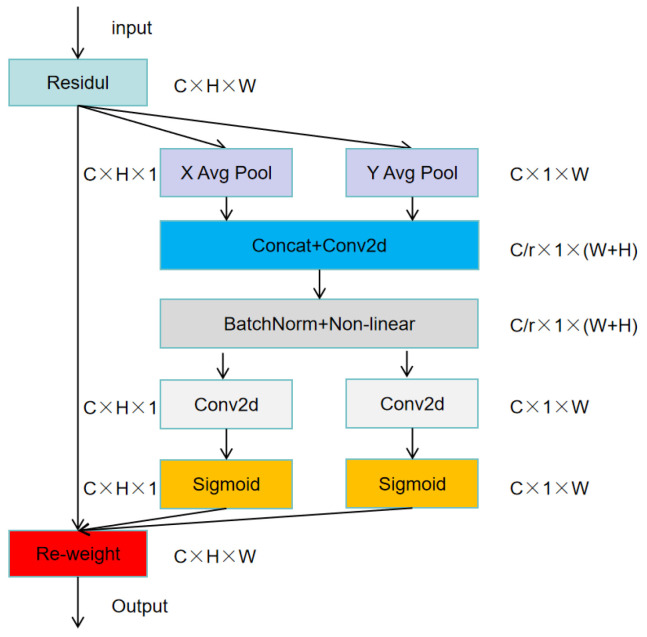
The structure of CA.

In addressing the challenge of detecting small cow ear tags within the dataset, the aforementioned improvements still exhibited limitations in their small target detection capabilities. To further enhance the model’s ability to detect small objects, we introduced CA at the end of the Backbone. Due to the lower resolution of feature maps at the network’s endpoint, placing CA at the end not only reduced the computational load but also maximized its effectiveness. This strategic placement integrated channel and spatial information, prompting the model to pay closer attention to the region containing the ear tag. Consequently, there was a discernible improvement in ear tag detection performance to a certain extent. The structure is shown in Figure 8.

Firstly, it performs global pooling on the input feature map in both horizontal and vertical directions, enabling the capture of long-range dependencies and positional information along the horizontal and vertical axes. The vectors from both directions are then concatenated, followed by convolution operations and batch normalization (BN) with a nonlinear activation. Subsequently, the feature map is split into horizontal and vertical segments, each undergoing convolution operations and then activated via a sigmoid function. Finally, the two resulting feature maps are multiplied element-wise by the input feature map, yielding a weighted feature map as the output.

This coordinate-based attention mechanism, by integrating both horizontal and vertical coordinate information, perceives not only channel information but also positional information, thus enhancing the network’s sensitivity to locational data. It enables the model to more effectively capture dependencies in the spatial dimension, facilitating the detection and localization of small objects in complex scenarios.

### 4.2. Ear Tag Number Detection

The detected ear tag backgrounds were quite complex, with some instances featuring interference, such as cow hair, rails, and wall corners. The images exhibited challenges such as angular deviations, distortion of the text, varying dimensions, and diverse fonts. Traditional character detection and segmentation methods require preprocessing steps such as perspective transformation and orientation correction before detection. Moreover, achieving real-time and accurate ear tag recognition is difficult when using these conventional approaches. Therefore, this study adopted DBNet to achieve fast and high-precision number detection.

**Figure 9:**
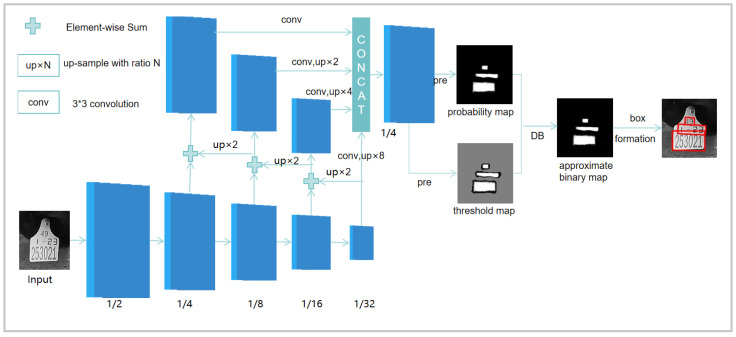
The structure of DBNet.

DBNet is a text detection algorithm based on segmentation and the concept of learnable thresholds, as illustrated in Figure 9. The input image underwent feature extraction using the Backbone, followed by feature fusion using the FPN structure. The network predicted probability maps and threshold maps of the text regions. The probability maps and threshold maps were then subjected to differentiable binarization to obtain an approximate binary image. Finally, a postprocessing step was applied to derive the bounding curves of the detected text.

The differentiable binarization computation employed an approximate step function to make the binarization process differentiable. By learning an adaptive threshold through a neural network, it achieved the segmentation of text regions. The definition of the approximate step function is as follows:

(1) $$B_{i,j}=\frac{1}{1+e^{-k(P_{i,j}-T_{i,j})}}$$

where $B_{i,j}$ represents the approximate binary image, k is the amplification factor, $P_{i,j}$ represents the probability map, and $T_{i,j}$ represents the threshold map.

### 4.3. Ear Tag Recognition

**Figure 10:**
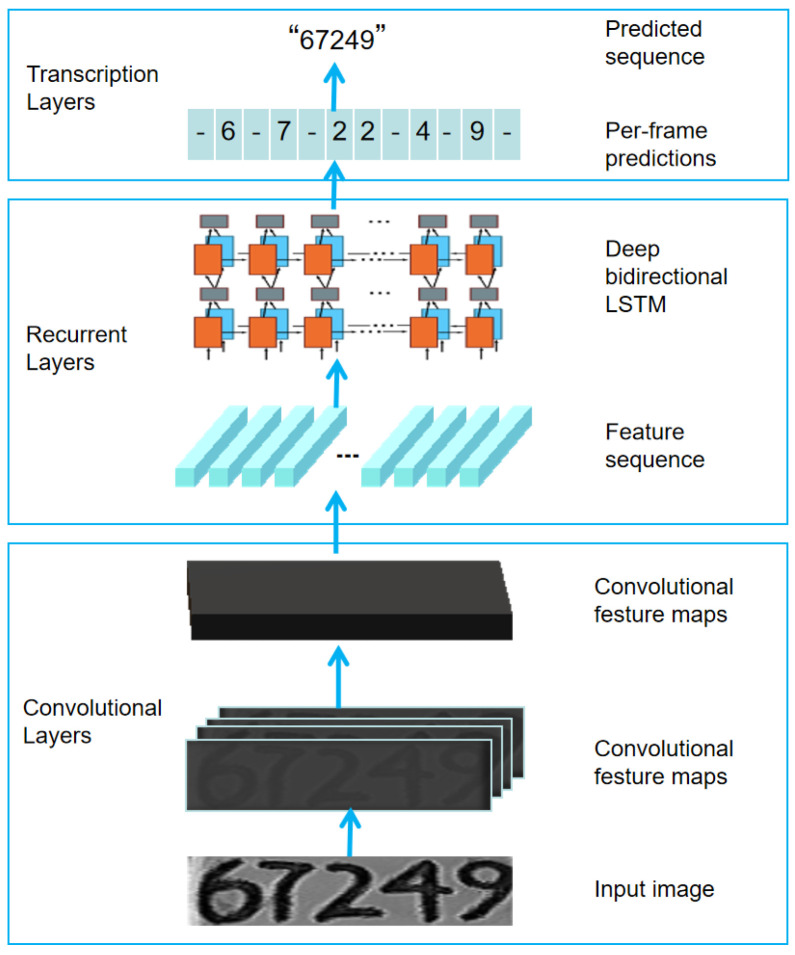
The structure of the CRNN.

The CRNN designed for ear tag number recognition comprised three components, as illustrated in Figure 10: convolutional layers, recurrent layers, and transcription layers. The convolutional layers executed feature extraction through convolution operations on the input image, yielding deeper-dimensional feature maps. Concurrently, the input images were mandated to have dimensions of W × 32, in which the height was fixed at 32, and the width could vary. Given the distinctive characteristics of ear tag numbers and experimental verification, we determined that configuring W as 320 resulted in a comparatively higher recognition accuracy. Consequently, the input image size was consistently set to 320 × 32.

The recurrent layers transformed the feature maps obtained from the convolutional layers into a sequence of features, which served as an input for the RNN to perform feature fusion. The RNN segment in this study involved a bidirectional long short-term memory (LSTM) structure. Compared to a regular recurrent neural network, it could handle longer sequences, conduct bidirectional predictions, enhance recognition accuracy, and address the issues of gradient vanishing and explosion during the training of long sequences.

The connectionist temporal classification (CTC) mechanism was utilized in the transcription layer by introducing the special character “-” as a blank placeholder to determine the beginning or end of a letter. In Figure 10, for instance, the input image with the label “67249” processed through the CRNN yielded a predicted sequence such as “-6-7-22-4-9-”. After CTC processing, it became “-6-7-2-4-9-”, and upon removing the special characters, the final prediction result was “67249”. This CTC processing mechanism addressed issues such as unequal input–output sequence lengths, a lack of alignment, and repetitive predictions, thus avoiding character-level segmentation. It achieved end-to-end prediction for sequences of variable lengths.

## 5. Experiment

### 5.1. Evaluation Metrics

To validate the effectiveness of the proposed model in the ear tag recognition process, we adopted several evaluation metrics, including precision (Equation (2)), recall (Equation (3)), average precision (AP) (Equation (4)), mean average precision (mAP) (Equation (5)), average prediction time per image (Time), model parameters (Parameters), F1_score (Equation (6)), and accuracy (Equation (7)).

Precision reflects the accuracy of the model in predicting positive samples, and it is defined as follows:

(2) $$Precision=\frac{TP}{TP+FP}$$

Recall denotes the proportion of predicted positive samples with respect to the actual positive samples; it measures the model’s ability to identify all relevant instances, and it is expressed in the following formula:

(3) $$Recall=\frac{TP}{TP+FN}$$

Average precision reflects the average precision at different recall levels. In the following formula, P represents precision, and R represents recall:

(4) $$AP=\int_{0}^{1}P\left(R\right)\;dR$$

The mean average precision (mAP) is the average of the average precision (AP) scores calculated for different categories when a model detects multiple classes. It serves as a comprehensive metric for evaluating the overall performance of a detection model. A higher mAP indicates better detection performance. Typically, mAP@0.5 is used to assess detection models, where 0.5 represents the threshold for the intersection over union (IoU) between predicted and actual bounding boxes. The definition of mean average precision is as follows, where N is the number of detection categories:

(5) $$mAP=\frac{1}{N}\sum_{i=1}^{N}AP_{i}$$

The F1_score provides a comprehensive measure of both precision and recall, and it represents the harmonic mean of precision and recall. It is defined according to the following formula:

(6) $$F1\_score=\frac{2\cdot Precision\cdot Recall}{Precision+Recall}$$

Accuracy represents the proportion of correctly predicted samples with respect to the total number of samples. In this study, it was used to evaluate the predictive performance of the ear tag recognition model:

(7) $$Accuracy=\frac{Correct\;Predictions}{Total\;Samples}$$

### 5.2. Experimental Setup

In this experiment, the ear tag detection, number detection, and ear tag recognition models were trained on a Linux operating system. The ear tag detection model was trained for 150 epochs with a batch size of 64, the number detection model (DBNet) was trained for 300 epochs with a batch size of 16, and the ear tag recognition model (CRNN) was trained for 300 epochs with a batch size of 128. All experimental datasets were divided into training, validation, and test sets in a ratio of 6:2:2. The specific hardware and software configurations used in the experiment are detailed in Table 1.

### 5.3. Experimental Results and Analysis

The YOLOv5 architecture consisted of four different-sized model configurations: small (s), medium (m), large (l), and extra-large (x). Initially, these four models, along with the Small-YOLOv5s proposed in this study, were trained using the cow ear tag detection dataset. An analysis was conducted based on precision, recall rate, mean average precision (mAP), single-image prediction time, and model parameter count to evaluate the detection performance of the different models. A comparison of the experimental results is presented in Table 2.

Firstly, an analysis of the YOLOV5 (s, m, l, and x) models was conducted. In Table 2, it can be observed that the inference speed per image gradually decreased, and the parameter count increased. YOLOV5s had the fewest parameters at 7,053,910, while YOLOV5x had the most parameters at approximately 87,198,694, an increase of about 80 million parameters compared to YOLOV5s. However, the four models exhibited similar precision, recall, and mAP@0.5. YOLOV5x had the highest mAP@0.5 at 91.9%, which was only 0.3% higher than that of YOLOV5s. Overall, these four models showed comparable performance of cow ear tag detection, with YOLOV5s being the most optimal. Therefore, we decided to use YOLOV5s as the baseline network structure for further improvements.

The precision of Small-YOLOV5s was 88.8%, the recall rate was 90.0%, and the mAP@0.5 was 92.5%. Compared to the values of YOLOV5s, the precision decreased by 1.3%, the recall rate increased by 1.5%, and the mAP@0.5 increased by 0.9%. Analyzing the inference speed and parameter count revealed that Small-YOLOV5s achieved an average reduction of 0.5 ms in the inference time per image compared to YOLOV5s. The parameter count decreased from 7,053,910 to 1,606,108, a reduction of 5,447,802 parameters.

The reason was that the lower-dimensional feature maps of Small-YOLOV5s significantly reduced the parameter count, leading to a noticeable increase in inference speed. The detection layer’s higher-resolution feature maps contained stronger positional information; enhancing the detection performance for small targets enabled more accurate detection of smaller ear tag regions, including those that might not have been labeled. Consequently, there was a slight decrease in precision. To further validate the effectiveness of Small-YOLOV5s in ear tag detection, ablation experiments on the improved part are presented in Table 3, where “-Conv” denotes the removal of the C3_9 module and Conv module from the Backbone.

The results indicated that YOLOV5s-Conv achieved a precision of 90.8% and mAP@0.5 of 92.1%, showing improvements compared to YOLOV5s. The parameter count significantly decreased, by 5,454,482, resulting in an increase in the inference speed by 0.3 ms. This suggested that removing the C3_9 module and Conv module from the sixth and seventh layers of the Backbone had a positive impact on small target detection.

After adding the CA in YOLOV5s-Conv, despite the increase in parameters by 6680, the model’s prediction speed improved by 0.2 ms. The recall increased by 2.2%, and mAP@0.5 improved by 0.4%. The analysis suggested that the addition of the CA facilitated the fusion of channel and spatial information, making the model more attentive to ear tag regions and enhancing detection performance.

To further validate the superiority of the CA in ear tag detection, a comparison was made with the Squeeze-and-Excitation (SE) [25] and Convolutional Block Attention Module (CBAM) [26], as shown in Table 4. The CA exhibited a 2.2% increase in recall, along with improvements of 0.5% and 1.5% in mAP@0.5 compared to SE and CBAM, respectively. Additionally, there were reductions in the parameters and prediction time, making the model more lightweight and flexible.

**Figure 11:**
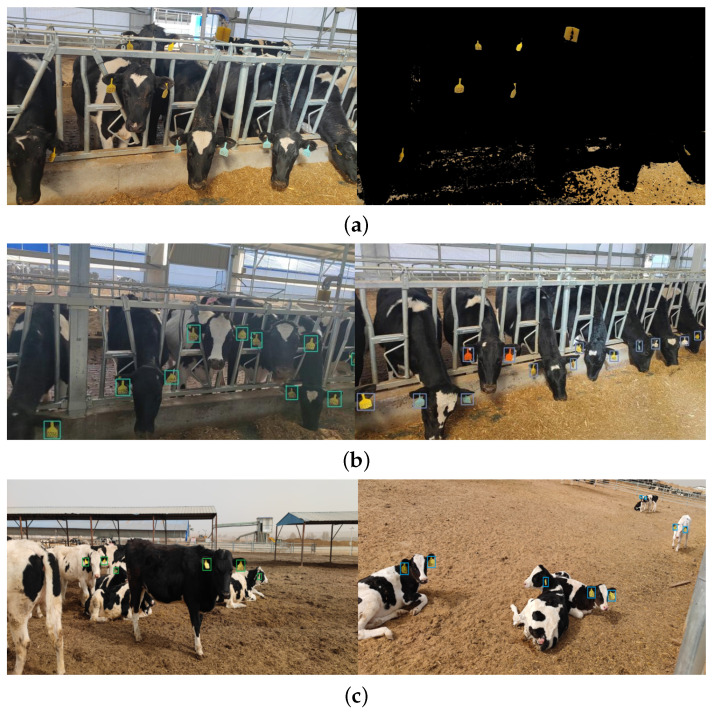
Comparison of cow ear tag detection results. (**a**) The results of ear tag detection using the color threshold method, with the original image on the left and the detection results on the right. (**b**,**c**) The detection results of cow ear tags in different scenarios using Small-YOLOV5s.

Currently, the existing method for ear tag detection employs the color threshold technique to segment and extract the region containing ear tags. The detection principle relies on discerning the target area based on its color disparity from the background. Initially, the image is converted into the HSV color space, followed by setting a specific color threshold range for detecting the desired targets. The efficacy of this approach is compared with that of Small-YOLOV5s in Figure 11 below.

When employing the color threshold method for ear tag detection with the color threshold set to the yellow range, it becomes apparent that, besides detecting yellow ear tags, it also identifies all yellow objects in the image, such as feed, causing some interference in ear tag detection. Furthermore, it is incapable of detecting ear tags of other colors in the image. In contrast, Small-YOLOV5s demonstrates the capability to simultaneously detect ear tags in various scenarios, under different lighting conditions, and with multiple cow poses, irrespective of tag color. It exhibits strong robustness in handling diverse conditions.

DBNet was trained using both three-channel RGB images and single-channel grayscale images. The performance in number detection was evaluated using the precision, recall, and F1_score, and the experimental results are presented in Table 5.

In the table above, it can be observed that, when the input image was a single-channel grayscale image, DBNet achieved a precision of 94.0%, a recall of 96.1%, and an F1_score of 95.0%. The performance in number detection was better than that in the case when the input image was a three-channel RGB image. The analysis suggested that, when the input was a single-channel grayscale image, the image structure and background were simpler, making it easier to extract features in the number region, leading to improved detection performance.

**Figure 12:**
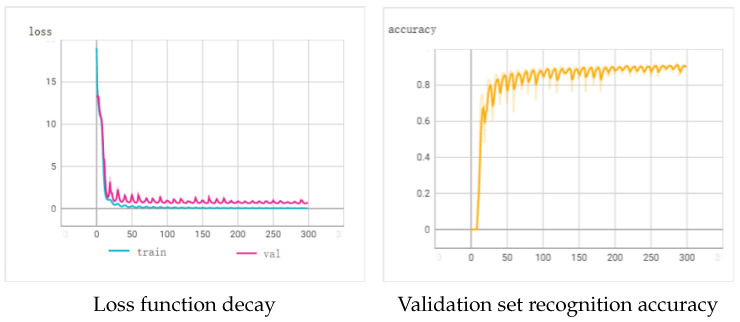
Loss decay and recognition accuracy in CRNN training.

The variation curve of the loss function and the recognition accuracy on the validation set during the training process of the CRNN ear tag recognition model are shown in Figure 12. After approximately 50 epochs, the loss function exhibited a converging trend, and beyond 200 epochs, the recognition accuracy for the validation set fluctuated around 92%. Finally, the model was tested on the test set, achieving a final recognition accuracy of 92.1%, with an average inference time of 0.62 ms per image.

### 5.4. Discussion

This study focused on the development of an automatic ear tag recognition system for dairy cows, which consisted of two main components: ear tag detection and ear tag recognition. To meet the practical requirements of real-world scenarios, we proposed Small-YOLOV5s by enhancing YOLOV5s to achieve a lightweight and easily deployable model with improved detection performance. A series of ablation experiments was conducted to validate our approach. The experimental results demonstrated that Small-YOLOV5s was well-suited for ear tag detection tasks, surpassing the limitations of traditional color thresholding methods that rely on single-color cues. Moreover, our model exhibited robustness against various disturbances, such as lighting conditions, backgrounds, and rotations.

During the ear tag recognition phase, this study initially employed DBNet for the detection of the number region and subsequently utilized a CRNN to recognize the ear tag number. The combined approach of DBNet+CRNN was better suited for accurately recognizing cow ear tag numbers against complex backgrounds. It enabled the simultaneous detection and recognition of multiple lines of text, eliminating the need for character-level segmentation. This method achieved coarse-grained end-to-end ear tag recognition, overcoming limitations associated with traditional image processing techniques and OCR recognition in terms of text region localization, segmentation, and recognition accuracy. Moreover, our experimental dataset comprised both handwritten and printed numbers on ear tags, demonstrating excellent performance in recognizing both types.

Although the research presented in this study demonstrated a high level of accuracy in cow ear tag recognition, there are certain limitations and shortcomings that need to be addressed. To enhance the composition of our experimental data, we primarily collected datasets from various angles using cell phones and high-resolution cameras, with a limited range of collection heights. However, it should be noted that actual barns are equipped with cameras with a fixed angle and height, which may impact the model’s performance.

When cows are at a certain distance from the camera, the ear tags in the images become smaller, and they appear relatively blurry, posing a significant challenge to ear tag recognition. Identification becomes difficult when dairy cow ear tags are obstructed by other objects or when multiple cows are in close proximity, leading to overlapping ear tags that are hard to differentiate. Additionally, this study employed a two-stage approach for ear tag recognition in which the numbered area was first detected and then recognized, resulting in an overall decrease in the recognition speed. Therefore, the further optimization and improvement of the model are necessary to enhance its speed and generalization ability so as to better meet real-world application requirements.

## 6. Conclusions

To address the development needs of modern dairy farming and tackle the issue of individual recognition in fine management, this study proposed a vision-based cow ear tag recognition method. Firstly, two self-constructed canonical experimental datasets (CEID-D and CEGD-R) were introduced, and they have been made freely available to the public. Subsequently, we improved the YOLOV5s model and proposed the lightweight Small-YOLOV5s for the detection of cow ear tags. Our experiments demonstrated that, compared with the original network, our model significantly reduced the number of parameters while enhancing the detection accuracy, and it was more lightweight and computationally efficient with an improved detection speed and better performance in small target detection. Finally, we employed DBNet+CRNN to achieve ear tag recognition with an accuracy of 92.1%.

To further enhance the performance in ear tag recognition, we will continue to optimize and refine the model while conducting corresponding experiments in future research endeavors. Moreover, we aim to broaden the scope of our investigation by incorporating individual tracking mechanisms for cows, integrating video-based ear identification techniques, and combining methods that identify individual movements and behaviors of cows. This integration will enable the real-time monitoring, tracking, positioning, behavior detection, and health analysis of cows, thus ultimately promoting both the well-being promotion of cattle farming practices and advancements in digital and intelligent farming industries.

## Figures and Tables

**Table 1:** Configuration of the experimental environment.

Term	Configurations
Operating System	CentOS Linux 7.7.1908
GPU	NVIDIA Quadro P5000 (NVIDIA Corporation, Santa Clara, CA, USA)
Memory	64 GB
Python	3.8.17
Pytorch	1.9.1
CUDA	11.2
CUDNN	10.0.130

**Table 2:** Experimental results for ear tag detection with different models.

Models	P (%)	R (%)	mAP@0.5 (%)	Time (ms)	Parameters
YOLOV5s	90.1	88.5	91.6	2.4	7,053,910
YOLOV5m	89.1	88.8	91.6	3.1	21,037,638
YOLOV5l	90.6	87.7	90.6	3.2	46,600,566
YOLOV5x	89.6	88.0	91.9	4.6	87,198,694
Small-YOLOV5s	88.8	**90.0**	**92.5**	**1.9**	**1,606,108**

**Table 3:** Results of comparative ablation experiments for Small-YOLOV5s.

Models	P (%)	R (%)	mAP@0.5 (%)	Time (ms)	Parameters
YOLOV5s	90.1	88.5	91.6	2.4	7,053,910
YOLOV5s-Conv	90.8	87.8	92.1	2.1	1,599,428
YOLOV5s-Conv+CA	88.8	90.0	92.5	1.9	1,606,108

**Table 4:** Comparison of the experimental results after the addition of CA, SE, and CBAM.

Models	P (%)	R (%)	mAP@0.5 (%)	Time (ms)	Parameters
YOLOV5s-Conv+SE	90.2	87.8	92.0	1.7	1,608,004
YOLOV5s-Conv+CBAM	89.5	87.8	91.0	2.0	1,608,102
YOLOV5s-Conv+CA	88.8	90.0	92.5	1.9	1,606,108

**Table 5:** Comparison of the experimental results of ear tag number detection.

Models	Precision (%)	Recall (%)	F1_Score (%)
DBNet (RGB)	93.5	94.8	94.1
DBNet (Gray)	94.0	96.1	95.0

## Data Availability

Data were obtained from Kaggle and are available at https://www.kaggle.com/datasets/fandaoerji/cow-eartag-detection-dataset and https://www.kaggle.com/datasets/fandaoerji/cow-eartag-recognition-dataset (accessed on 20 January 2024 with the permission of Kaggle).
